# Risk and Prognosis of Secondary Bladder Cancer After Radiation Therapy for Rectal Cancer: A Large Population-Based Cohort Study

**DOI:** 10.3389/fonc.2020.586401

**Published:** 2021-01-25

**Authors:** Xu Guan, Ran Wei, Runkun Yang, Zhao Lu, Enrui Liu, Zhixun Zhao, Haipeng Chen, Ming Yang, Zheng Liu, Zheng Jiang, Xishan Wang

**Affiliations:** ^1^ Department of Colorectal Surgery, National Cancer Center/National Clinical Research Center for Cancer/Cancer Hospital, Chinese Academy of Medical Sciences and Peking Union Medical College, Beijing, China; ^2^ The Second Affiliated Hospital of Harbin Medical University, Harbin, China

**Keywords:** rectal cancer, radiation therapy, secondary bladder cancer, prognostic factor, overall survival, cancer specific survival

## Abstract

**Background:**

Although radiation therapy (RT) improves local control for rectal cancer (RC), the long-term risks from RT, including development of a secondary malignancy, are controversial. The risk and prognosis of secondary bladder cancer (SBC) in RC patients undergoing RT have not been adequately studied. Our goal is to investigate the impact of RT on the risk of developing SBC and assess their survival outcomes.

**Methods:**

This large population-based study included RC patients as their initial primary cancer from nine registries of the Surveillance, Epidemiology and End Results (SEER) database between 1973 and 2015. The cumulative incidence of SBC was assessed by using Fine and Gray’s competing risk regression. The standardized incidence ratio (SIR) was used to compare the incidence of SBC in RC survivors to the US general population. The Kaplan-Meier method was used to evaluate the 10-year overall survival (OS) and 10-year cancer specific survival (CSS) for patients with SBC.

**Results:**

Of 74,646 RC patients, 24,522 patients were treated with surgery and RT and 50,124 patients were treated with surgery alone. The incidence of SBC was 1.85% among patients who received RT and 1.24% among patients who did not. The incidence of SBC in RC patients who received RT was higher than the US general population (SIR, 1.35; 95% CI, 1.19-1.53, P<0.05), and decreased with increasing age at diagnosis, and increased with time since diagnosis. In competing risk regression analysis, undergoing RT was associated with a higher risk of SBC (hazard ratio [HR], 1.443, 95% confidence interval [CI], 1.209-1.720; P<0.001). The results of the dynamic SIR for SBC revealed that a slightly increased risk of SBC was observed after RT in the early latency, and was significantly related to the variations of age at RC diagnosis and decreased with time progress. The 10-year OS and CSS among SBC patients after RT were comparable to SBC patients after NRT.

**Conclusion:**

Radiation was associated with an increased risk of developing SBC in RC patients, and special attention should be paid to the surveillance of these patients.

## Introduction

Total mesorectal excision and (neo) adjuvant radiation therapy (RT) has been considered as the standard treatment regimen for locally advanced rectal cancer (RC), with superior local control when compared with surgery alone (1, 2). However, because of ionizing radiation, RT has been associated with several late side effects for long-term RC survivors (3). One of the most serious late side effects, the increased risk of occurring a radiation-induced second primary cancer (SPC) is controversial (4–6).

SPC is commonly seen in long term cancer survivors, and approximately 8% of patients with common cancers developed a second malignancy (7). Risk factors for the development of SPC are multifactorial, including normal aging, genetic predisposition, environmental and lifestyle risk factors, and treatment for initial primary cancer (5, 7, 8). Some of these risk factors could be partially avoidable, through methods such as adopting a healthy lifestyle and getting appropriate cancer treatment.

Although several studies have assessed the role of RT in the development of SPC for RC patients, the conclusions are inconsistent regarding the role of RT (9, 10). Warschkow et al. found a decreased overall risk of second malignancies after pelvic radiation attributed to decrease in prostate cancer following pelvic radiation (11). Therefore, the prostate gets a significant dose but had a lower chance for cancer. Furthermore, whether RT has an adverse effect on the survival outcome of SBC has very important prognostic and therapeutic implications, but no study has confirmed this issue. Therefore, we used the National Cancer Institute’s Surveillance, Epidemiology, and End Results (SEER) database to investigate the impact of RT for RC patients on the subsequent risk of occurring an SBC and to evaluate their long-term prognosis.

## Methods

### Database and Study Population

We identified patients diagnosed with histologically confirmed RC as their initial primary cancer from nine registries of the SEER program between January 1973 and December 2015. The RC (C20.9) and rectosigmoid cancer (C19.9) were included according to The 3rd Edition of International Classification of Diseases for Oncology (ICD-O-3). Localized and regional stage as defined by SEER was chosen for analysis. Exclusion criteria included patients where RC was not their first primary cancer, age younger than 20 years, survival less than 1 year after RC diagnosis, no rectal cancer surgery, distant metastases, and missing data on radiation, surgery, age, tumor stage, race and follow-up information. This study has been approved by the Ethics Committee of Cancer Hospital, Chinese Academy of Medical Sciences.

### Treatment Interventions and Outcomes

RC patients were classified into two groups according to initial treatment modality. The RT group was composed of RC patients who received surgery and (neo)adjuvant external-beam RT, and the no RT (NRT) group was composed of patients who received surgery alone. To avoid bias caused by different modalities of RT, patients who received brachytherapy or combination RT were excluded from our analysis.

The primary outcome of the present study was to investigate the risk of development of a SBC more than one year after treatment of RC. The SEER program avoids the inclusion of recurrent disease of RC according to the ICD-O-3 guidelines. The follow-up for SBC started at 1 year after the initial treatment of RC and ended at the date of all-cause death, diagnosis of SBC, or reaching 30 years follow-up, whichever occurred first.

The secondary outcome was to evaluate the 10-year overall survival (OS) and 10-year cancer specific survival (CSS) of SBC. The definition of OS was the time from SBC diagnosis to the date of all-cause death, and the definition of CSS was the time from SBC diagnosis to the date of SBC-cause death. The survival analysis was performed by using case-control design, in which each SBC patient who received RT was compared with each SBC patient who did not received RT or with five patients diagnosed with only primary BC (OPBC). The definition of OPBC was the patient diagnosed with only BC, without any other malignancies diagnosed during their lifetime.

### Statistical Methods

Fine-Gray competing risk regression analysis was performed with SBC as an event and a non-SBC or all-cause death were considered competing events to estimate the hazard ratios (HRs) and 95% confidence intervals (CIs) of SBC occurrence after RC. The multivariable risk model was built by using a backward selection procedure with variables with P values less than 0.05 (two sided) in univariable analyses, which were considered statistically significant and included in multivariable analyses. This analysis was performed with R software (version 3.5.3).

Furthermore, we calculated the standardized incidence ratio (SIR) and 95% CIs with Poisson regression analysis. The definition of SIR in our study was the ratio of observed incidence SBC among RC patients to the incidence of BC in the US general population. SIR was adjusted for sex, age at RC diagnosis, and the calendar year of RC diagnosis. To assess the dynamic risks and incidence for SBC, the SIRs were stratified by age at RC diagnosis, latency time since RC diagnosis and year of RC diagnosis. The SIR was calculated with SEER*Stat 8.3.6.

The Kaplan-Meier method was used to calculate the 10-year OS for SBC and OPBC, and P values were calculated with the log-rank test. To reduce possible bias for survival comparison, we used propensity score matching (PSM) to match the cases and controls by using variables of sex, age at BC diagnosis, race, year of BC diagnosis, stage of BC, tumor grade, and treatment for BC. These analyses were performed using R software (version 3.5.3).

## Results

### Patient Characteristics

**Figure 1:**
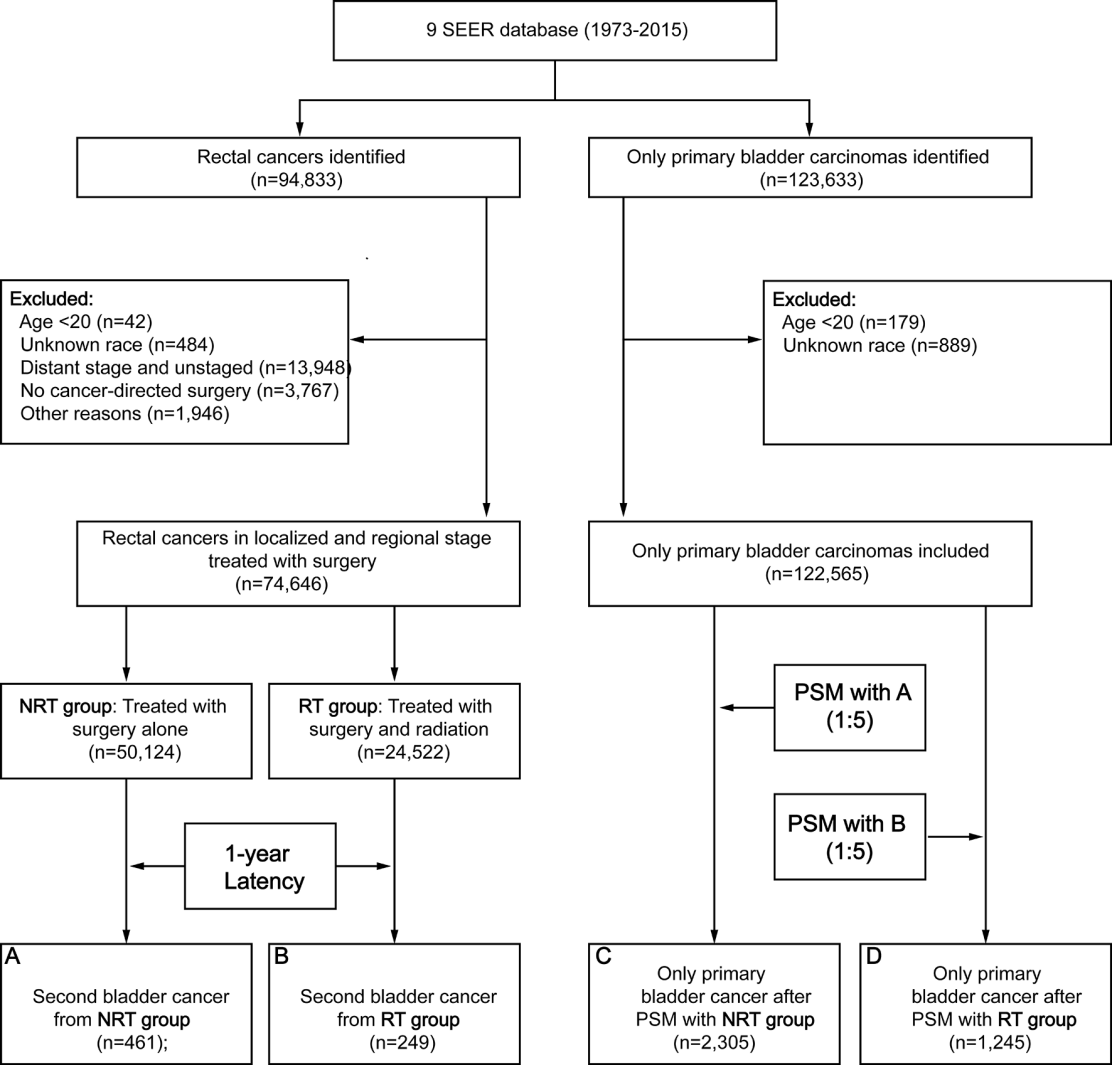
Flow diagram. RT, radiation therapy; NRT, no radiation therapy; SEER, Surveillance, Epidemiology and End Results; PSM, propensity score matching.

A total of 74,646 RC patients were included in our analysis, 24,522 patients received RT and 50,124 patients did not receive RT (**Figure 1**). After one-year latency from RC diagnosis, 249 patients in RT group and 461 patients in the NRT group developed an SBC. The median follow-up time was 69 (35-135) months in the RT group and 85 (40-164) months in the NRT group. Compared with patients in the NRT group, patients in the RT group presented with younger, higher proportion of male sex, poor differentiation, regional stage, and **mucinous** tumor, with p< 0.05. In the RT group, more patients received chemotherapy compared with patients in the NRT group, with p< 0.05. The baseline characteristics of RC patients who developed an SBC were similar to RC patients. The detailed information of RC patients and RC patients who developed an SBC by treatment modality were shown in **Table 1**.

**Table 1:** Comparison of baseline characteristics of all RC patients and those who developed SBC by treatment modality.

Characteristic	Surgery alone(n=50,124)	Surgery with RT(n=24,522)	P-value	Surgery alone(n=461)	Surgery with RT(n=249)	P-value
Median age at RC diagnosis, (IQR), years	66 (57-75)	62 (53-70)	<0.001^a^	68 (62-75)	65 (58-73)	<0.001^a^
Age at RC diagnosis, No. (%), years			<0.001^b^			0.008^b^
20-49	4,921 (9.8)	4,211 (17.2)		15 (3.3)	17 (6.8)	
50-69	24,840 (49.6)	13,870 (56.5)		241 (52.3)	146 (58.6)	
≥ 70	20363 (40.6)	6441 (26.3)		205 (44.4)	86 (34.6)	
Median year of RC diagnosis (IQR)	1993 (1983-2003)	2000 (1991-2008)	<0.001^a^	1989 (1983-1998)	1995 (1989-2001)	<0.001^a^
Year of RC diagnosis, No. (%)			<0.001^b^			<0.001^b^
1975-1984	14,092 (28.1)	2,604 (10.6)		140 (30.4)	39 (15.7)	
1985-1994	12,951 (25.8)	5,710 (23.3)		154 (33.4)	85 (34.1)	
1995-2004	11,772 (23.5)	7,500 (30.6)		127 (27.5)	92 (36.9)	
≥ 2005	11,309 (22.6)	8,708 (35.5)		40 (8.7)	33 (13.3)	
Sex, No. (%)			<0.001^b^			<0.001^b^
Female	23,140 (46.1)	9,493 (38.7)		97 (21)	53 (21.3)	
Male	26,984 (53.9)	15,029 (61.3)		364 (79)	196 (78.7)	
Race, No. (%)			0.003^b^			0.309^b^
White	41,825 (83.4)	20,365 (83)		432 (93.7)	227 (91.2)	
Black	3,555 (7.1)	1,659 (6.8)		14 (3)	8 (3.2)	
Other	4,744 (9.5)	2,498 (10.2)		15 (3.3)	14 (5.6)	
Tumor grade, No. (%)			0.001^b^			0.001^b^
Grade I/II	34,238 (68.3)	17,921 (73.1)		314 (68.1)	184 (73.9)	
Grade III/IV	4,416 (8.8)	4,035 (16.5)		42 (9.1)	40 (16.1)	
Unknown	11,470 (22.9)	2,566 (10.4)		105 (22.8)	25 (10)	
Tumor stage, No. (%)			<0.001^b^			<0.001^b^
Localized	35,064 (70)	7,099 (28.9)		348 (75.5)	90 (36.1)	
Regional	15,060 (30)	17,423 (71.1)		113 (24.5)	159 (63.9)	
Tumor histology, No. (%)			<0.001^b^			<0.001^b^
Adenocarcinoma	47,596 (95)	22,197 (90.5)		442 (95.9)	222 (89.2)	
Mucinous tumor	1,964 (3.9)	1,932 (7.9)		17 (3.7)	27 (10.8)	
Other	564 (1.1)	393 (1.6)		2 (0.4)	0 (0)	
Tumor size, No. (%), cm			<0.001^b^			<0.001^b^
< 2	3,460 (6.9)	743 (3)		17(3.7)	1 (0.4)	
≥ 2	5,839 (11.6)	7,213 (29.4)		23(5)	38 (15.3)	
Unknown	40,825 (81.5)	16,566 (67.6)		421(91.3)	210 (84.3)	
Chemotherapy, No. (%)			<0.001^b^			<0.001^b^
No	46,321 (92.4)	6,089 (24.8)		433 (93.9)	86 (36.5)	
Yes	3,803 (7.6)	18,433 (75.2)		28 (6.1)	163 (63.5)	
Median follow-up time of RC, (IQR), months	85 (40-164)	69 (35-135)	<0.001^a^	87 (41-159)	93 (41-162)	<0.001^a^

P-value was calculated using the Mann-Whitney U test (^a^) for continuous variables and χ^2^ test (^b^) for categorical variables.

RC, rectal cancer; RT, radiation therapy; NRT, no radiation therapy; IQR, interquartile ratio; SBC, secondary bladder cancer.

### Cumulative Incidence and SIR of SBC

**Figure 2:**
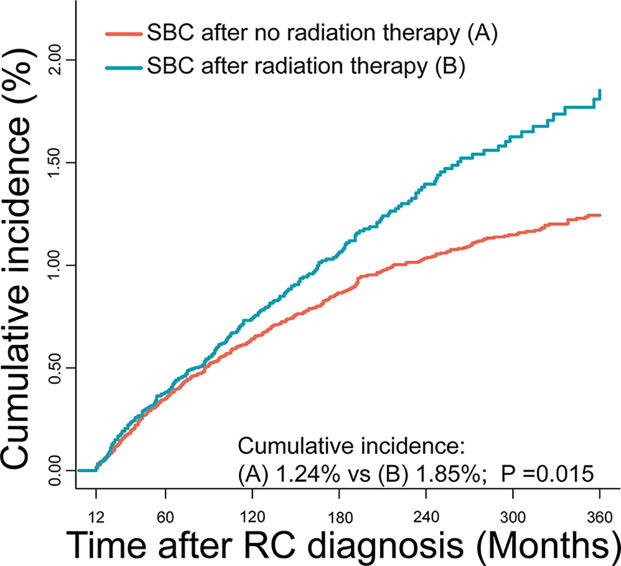
Comparisons of cumulative incidence of secondary bladder cancer (SBC) between patients who received radiation therapy (RT) and patients who did not receive RT. P values were calculated with the Gray test.

The cumulative incidence for developing SBC was 1.38% in RC survivors during 30 years of follow-up. The cumulative incidence of RC survivors developing SBC that received RT (1.85%) was higher than RC survivors developing SBC that did not receive RT (1.24%), with adjusted P=0.015 (**Figure 2**). Then, we calculated the SIRs to assess the incidence risk of SBC. The incidence risk of SBC in RC patients who received RT was higher than the US general population (SIR, 1.35; 95% CI, 1.19-1.53, P<0.05; **Table 2**). However, the incidence risk of SBC in patients who did not receive RT was similar to the US general population (SIR, 0.92; 95% CI, 0.84-1.01, P>0.05; **Table 2**).

**Table 2:** Standardized Incidence Ratio of Secondary Bladder Cancer by Age at RC Diagnosis, Latency and Year of RC Diagnosis.

Characteristic	(RT vs US general population)		(NRT vs US general population)	
	Adjusted SIR (95% CI)	P-value	Adjusted SIR (95% CI)	P-value
Secondary bladder cancer	1.35 (1.19-1.53)	p<0.05	0.92 (0.84-1.01)	–
Latency, Months				
12-59	1.33 (1.07-1.65)	p<0.05	1.05 (0.90-1.23)	–
60-119	1.24 (0.96-1.57)	–	0.9 (0.75-1.07)	–
120-239	1.42 (1.12-1.78)	p<0.05	0.84 (0.70-1.00)	–
240-359	1.51 (0.91-2.36)	–	0.82 (0.59-1.12)	–
Age at RC diagnosis, years				
20-49	1.93 (1.15-3.05)	p<0.05	0.77 (0.43-1.27)	–
50-69	1.34 (1.14-1.58)	p<0.05	0.9 (0.79-1.01)	–
≥ 70	1.29 (1.03-1.60)	p<0.05	0.97 (0.84-1.11)	–
Year of RC diagnosis				
1975-1984	1.80 (1.30-2.43)	p<0.05	0.87 (0.73-1.02)	–
1985-1994	1.39 (1.11-1.72)	p<0.05	0.90 (0.76-1.05)	–
1995-2004	1.30 (1.05-1.60)	p<0.05	1.00 (0.83-1.19)	–
≥ 2005	1.07 (0.74-1.50)	–	1 (0.71-1.36)	–

SIR was defined as the ratio of the number of observed secondary bladder cancer (SBC) cases among rectal cancer (RC) survivors to the expected number of cases in the US general population and was stratified by age at RC diagnosis and calendar year of RC diagnosis. A determination of the statistical significance of SIRs was based on a P<0.05 (two sided). 95% confidence intervals were calculated by Poisson exact methods. The background incidence of SBC was derived from data provided by the SEER database.

RC, rectal cancer; RT, radiation therapy; NRT, no radiation therapy; SIR, standardized incidence ratio; SBC, secondary bladder cancer; BC, bladder cancer.

### Risk of RT for Developing SBC

The variables presented in **Table 1** were selected to assess the risk of developing SBC in univariable competing risk regression (**Table 3**), and the variables including age at RC diagnosis, sex, race, year at RC diagnosis, tumor stage, tumor histology, and RT could significantly influence the risk of developing SBC in univariable analysis (with P< 0.05).

**Table 3:** Univariable and Multivariable Competing Risk Regression Analysis of Risk of Developing SBC in RC Patients.

Characteristic (OS)	Univariable analysis		Multivariable analysis	
	HR (95%Cl)	P-value	HR (95%Cl)	P-value
Age at RC diagnosis, per year	1.014 (1.010-1.020)	<0.001	1.017 (1.011-1.023)	<0.001
Year of RC diagnosis, per year	0.992 (0.986-0.999)	0.022	0.991 (0.984-0.998)	0.016
Sex				
Female	1		1	
Male	2.960 (2.470-3.550)	<0.001	3.030 (2.530-3.650)	<0.001
Race				
White	1		1	
Black	0.436 (0.285-0.666)	<0.001	0.508 (0.331-0.780)	<0.001
Other	0.421 (0.290-0.611)	<0.001	0.432 (0.298-0.607)	<0.001
Grade				
Grade I/II	1			
Grade III/IV	0.990 (0.783-1.250)	0.930		
Unknow	0.953 (0.786-1.160)	0.620		
Tumor stage				
Localized	1		1	
Regional	0.821 (0.706-0.955)	0.011	0.716 (0.608-0.842)	<0.001
Tumor histology				
Adenocarcinoma	1		1	
Mucinous tumor	1.140 (0.840-1.550)	0.400	1.129 (0.831-1.533)	0.440
Other	0.217 (0.041-0.870)	0.031	0.235 (0.058-0.944)	0.041
Tumor size (cm)				
<2	1			
≥2	0.906 (0.535-1.530)	0.710		
Treatment strategy				
Surgery alone	1			
Surgery with chemotherapy	1.030 (0.871-1.210)	0.740		
Treatment strategy				
Surgery alone	1		1	
Surgery with radiotherapy	1.210 (1.040-1.410)	0.015	1.443 (1.209-1.720)	<0.001

Fine-Gray competing risk regression analyses were used to calculate the hazard ratios (HRs) and 95% confidence intervals (CIs) for SBC in rectal cancer (RC) patients treated with RT versus patients not treated with RT. Covariables that are significant in univariable competing risk regression analysis (P<0.050) are included in the multivariable analysis.

HR, hazard ratio; CI, confidence interval; RC, rectal cancer; SBC, secondary bladder cancer.

**Figure 3:**
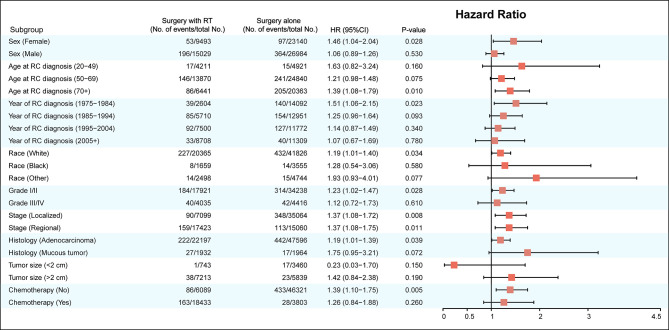
Subgroup analyses of competing risk regression for the risk of developing secondary bladder cancer.

The factors including age at RC diagnosis, sex, race, year at RC diagnosis, tumor stage, tumor histology, and RT were also associated with a higher risk of developing SBC in multivariate analysis (with P< 0.05; **Table 3**). In the final multivariable analysis, RT was an independent risk factor of developing SBC in RC survivors (HR, 1.443; 95% CI, 1.209-1.720; adjusted P< 0.001). In addition, subgroup analyses were performed to further evaluate the risk of developing SBC by competing risk regression. We found that the increased risk associated with RT was noted in most subgroups but not all were statistically significant (**Figure 3**).

### Dynamic Incidence Risk for SBC

**Figure 4:**
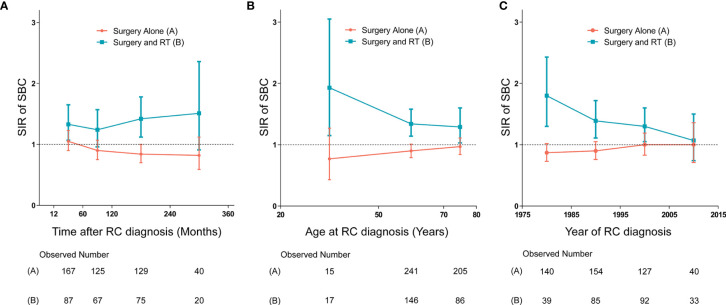
**(A)** Dynamic standardized incidence ratio (SIR) for secondary bladder cancer (SBC) in latency-SIR plot; **(B)** Dynamic SIR for SBC in age-SIR plot; **(C)** Dynamic SIR for SBC in diagnosis time-SIR plot. **(A–C)** SIRs of developing SBC in patients treated with radiation therapy (RT) versus the US general population are plotted, as well as patients treated without RT versus the US general population, and the incidence in the background US population is represented by the gray line (at y=1). The detailed data of SIRs are shown in the **Supplementary Data**. HR, hazard ratio; RC, rectal cancer; RT, radiation therapy; SIR, standardized incidence ratio; SBC, secondary bladder cancer.

We established three dynamic SIR plots based on time after RC diagnosis, age at RC diagnosis and year of RC diagnosis, to further evaluate the dynamic incidence risk of SBC for RC patients treated with and without receiving RT. In dynamic latency-SIR plot, significant incidence change of SBC with longer follow-up after RC diagnosis was only observed for patients treated with RT (**Figure 4A**). In dynamic age-SIR plot, decrease in risk of SBC was observed in different age groups at RC diagnosis only for patients treated with RT (**Figure 4B**). The younger patients treated with RT had a higher risk compared to older patients, which is compared with the US general population in the matching age group, although older patients are more likely to develop bladder cancer (**Figure 4B**). In dynamic diagnosis time-SIR plot, compared with the background incidence rate of SBC, a decrease in the risk of SBC was observed in patients with RC treated with surgery and RT. And this risk reaching baseline rates was observed for patients diagnosed with RC in 2005-2015 (**Figure 4C**).

### Survival Outcome of SBC

**Figure 5:**
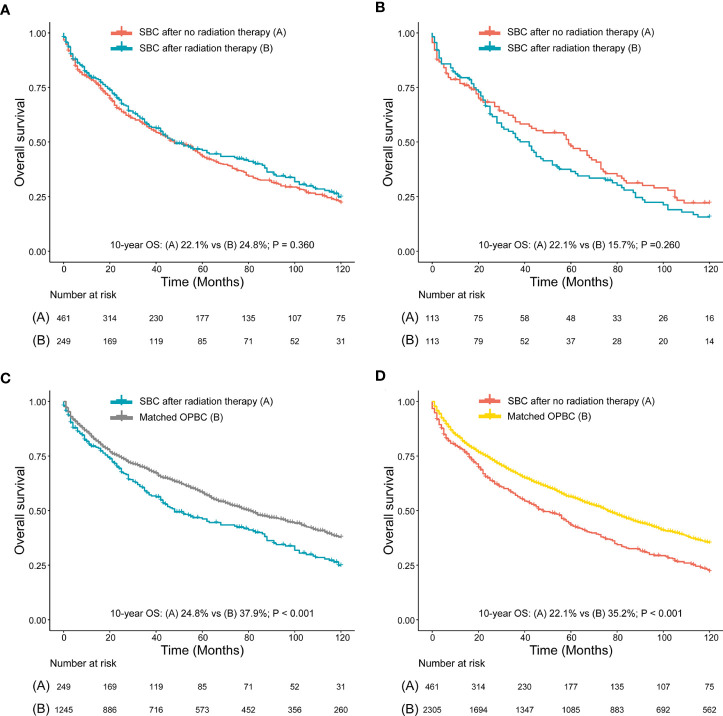
**(A)** Survival comparison between rectal cancer (RC) patients who developed secondary bladder cancer (SBC) after radiation therapy (RT) and RC patients who developed SBC after no RT (NRT) (before PSM); **(B)** Survival comparison between RC patients who developed SBC after RT and RC patients who developed BC after NRT (after PSM); **(C)** Survival comparison between RC patients who developed SBC after RT and patients with only primary bladder cancer (OPBC); **(D)** Survival comparison between RC patients who developed SBC after NRT and patients with OPBC. **(B)** RC patients who developed SBC after RT and RC patients who developed SBC after NRT were matched by PSM at a ratio of 1:1. **(C, D)** are case-control comparisons, RC patients who developed SBC (cases) versus patients with OPBC (controls), with a PSM ratio of 1:5 for SBC versus OPBC. The variables matched for PSM included age at SBC diagnosis, year of SBC diagnosis, race, stage of SBC and type of treatment for SBC. The detailed patient characteristics of OPBC before and after PSM are shown in the **Supplementary Data**. HRs were calculated using Cox regression. HR, hazard ratio; RC, rectal cancer; RT, radiation therapy; CI, confidence interval; SIR, standardized incidence ratio; SBC, secondary bladder cancer; OPBC, only primary bladder cancer.

We compared survival between patients with SBC after RT and NRT, and we found no significant differences of 10-year OS and 10-year CSS observed between patients who developed SBC after RT and patients who developed SBC after NRT, both before PSM (10-year OS, 22.1% vs 24.8%, P=0.360; 10-year CSS, 68.7% vs 74.7%, P=0.560; **Figure 5A**, **Supplementary Figure 1A**) and after PSM (10-year OS, 22.1% vs 15.7%, P=0.260; 10-year CSS, 71.9% vs 67.8%, P=0.260; **Figure 5B**, **Supplementary Figure 1B**), which may suggest that patients could have a higher risk of death but not due to bladder cancer within the 10-year follow-up. To further evaluate the survival outcomes of SBC, we matched the OPBC with SBC by using PSM. Compared with matched population controls with OPBC, significantly difference of 10-year OS was observed between patients developed SBC after RT and matched OPBCs (10-year OS, 24.8% vs 37.9%, P<0.001; 10-year CSS, 74.7% vs 71.0%, P=0.560; **Figure 5C**, **Supplementary Figure 1C**), and significant survival difference of 10-year OS was observed in patients without RT compared with matched OPBC (10-year OS, 22.1% vs 35.2%, P<0.001; 10-year CSS, 68.7% vs 69.3%, P=0.880; **Figure 5D**, **Supplementary Figure 1D**). Detailed information on OPGMs and survival analyses were shown in the **Supplementary Table 2, 3**, and **Supplementary Figure 1**.

## Discussion

To our knowledge, this is the first large population-based study to comprehensively assess the risk of developing SBC in RC survivors and to evaluate the survival outcomes of SBC. The cumulative incidence of SBC in RC patients who received RT was higher than those who did not receive RT, and RT was considered as an independent risk factor for SBC occurrence in RC patients. Second, the incidence of SBC in RC patients who received RT was higher than the US general population, and the incidence of SBC after RT decreased with diagnosis time and increased with latency period. The younger patients treated with RT had a higher risk compared to older patients, which is compared with the US general population in matching age group. Third, no survival differences were observed for SBC after RT compared with those without RT.

In literature, previous studies evaluating the risk of SBC after RT treatment for RC patients presented conflicting results (9–14). Several reasons could affect the interpretation of results, including sample size of cohort population, length of follow-up duration, latency period selection, and study methodology. Birgisson et al. has performed a study to observe the occurrence of SPC in RC patients treated with RT based on the participants from Uppsala Trial and Swedish Rectal Cancer Trial, and the results showed that both pre- and postoperative RT could not lead to a higher risk of developing SBC in RC patients. Due to the fact that only 12 SBCs were observed in this study, the sample size led to the limited statistical power for the conclusion (9). Furthermore, Wiltinik et al. performed a pooled trial cohort study including more than 2,500 patients with pelvic cancers, those who were treated with external-beam RT or vaginal brachytherapy had no increased probability of developing an SBC than patients who underwent surgery alone (10). Wang et al. used Taiwan’s National Health Insurance Research Database to evaluate the risk of SPC after pre- or postoperative RT in RC survivors. They found that preoperative RT had no increase in SBC, but the postoperative RT could increase risk of developing SBC (14). In our analysis, we found that RC patients who received RT had an increased risk of developing SBC compared with those who did not receive RT. The major strength of our study is the large sample size and the long-term follow-up of up to 30 years, which presented higher statistical power for the conclusion than previous studies.

The statistical consideration is very critical for the interpretation of results. In previous studies, Cox regression is preferred to assess the risk of SPC after RT treatment for RC patients (11, 13, 15, 16), but this approach might lead to a potential statistical bias because of a very small proportion of SBC occurrence in RC survivors. In our study, we used Fine-Gray competing risk regression to evaluate the risk of developing SBC. Compared with other statistical methods, competing risk regression in our study could adequately assess the risk of developing SBC against the competing events including development of a non-SBC second primary malignancy or all-cause death. In addition, we evaluated the SIR to compare the incidence of SBC in RC patients to the US general population, which could further suggest the influence of RT on the incidence of SBC.

In our study, we innovatively evaluated the dynamic incidence of SBC based on the latency period, age at RC diagnosis, and year of RC diagnosis. For RC survivors after treatment of RT, there was no obvious increased incidence of SBC in the early latency, but the incidence of SBC generally increased and the highest incidence of SBC was found after a latency of over 20 years. This finding indicated that long-term follow-up is necessary for the detection of SBC. Considering the influence of the age of RC patients on the risk of SBC, a tendency of decreased incidence for SBC was observed with increasing age at RC diagnosis, which suggested that young RC patients who underwent RT were at a higher risk of SBC compared with elderly RC patients. The possible reason contributing to the increased risk in young RC patients might be that young patients could have a long life expectancy which increased their likelihood of detection of SBC. With the improvements of RT treatment for RC, we found that the incidence of SBC gradually decreased from 1975-1984 to 2005-2015. During the study period from 1975 to 2015, radiation treatment modality has shifted with a trend toward hypofractionation, which may influence the incidence of SBC in more recent years. Besides, more precise radiotherapy target area formulation with time progress can further reduce the extra radiation exposure for the surrounding tissues and organs around the tumor.

Up to now, no studies have reported the prognosis of RT-related SBC. This is a very important clinical issue for SBC, because potential heterogeneity of RT-related SBC might be presented compared with non-RT-related SBC. To better address this issue, we performed survival analyses to compared prognosis of SBC after RT to those without RT and to matched OPBC. However, no survival differences were observed between SBC with and without RT. Although development of RT-related SBC is probably due to the induction of different genetic signaling pathways after radiation exposure which might be different from the normal signaling pathway of OPBC, the varied genetic phenotype of RT-related SBC is not resistant to standard treatment for OPBC.

### Limitations

The main limitations of the present study were as follows. First, a lack of randomization of the initial treatment for RC may contribute to potential biases. However, the occurrence of SBC may not only be associated with radiation exposure but may also be affected by other crucial risk factors, such as smoking history, genetic background, environmental factors, and other cancer-related treatments. These unmeasured covariates are likely related to both the primary cancer as well as the development of a second primary malignancy and, as such all influencing factors between the two treatment types could not be balanced. Instead, we adjusted all confounding risk factors using a multivariable risk competing model to reduce the potential effect of bias associated with the lack of randomization. Second, we could not determine the effect of dose, fractionation, and timing of radiation on the risk of SBC because of the lack of information on RT in the SEER database. However, the relatively homogenous treatment approach for the large observation population has been identified in the SEER database. Third, SEER database records only the initial treatment information for RC, and whether the delayed RT is performed in subsequent treatment is unknown. which therefore could lead to an underestimate of the actual risk of SBC associated with RT.

## Conclusion

Radiation for RC was associated with an increased risk of SBC. More attention should be paid to the surveillance of SBC in RC patients. The strengths of the present study include a long follow-up period to discover potential SBC as well as a large observation population with relatively homogenous treatment exposure identified from the SEER database. Furthermore, several methodologies were used together to obtain strong evidence of an increased risk of developing SBC in RC patients who received RT, and the risk of SBC was evaluated from several dimensions, which together could provide a more valuable reference for the treatment and follow-up of SBC in RC patients after RT.

## Data Availability Statement

Publicly available datasets were analyzed in this study. This data can be found here: Surveillance, Epidemiology, and End Results (SEER) database (https://seer.cancer.gov/).

## Author Contributions

Conception and design: XG, RW, ZJ, XW. Collection and assembly of data: XG, RW, RY, ZLu, MY, ZJ, XW. Data analysis and interpretation: XG, RW, EL, ZZ, HC, ZLiu, ZJ, XW. Manuscript writing, final approval of manuscript, accountable for all aspects of the work: All authors. All authors contributed to the article and approved the submitted version.

## Conflict of Interest

The authors declare that the research was conducted in the absence of any commercial or financial relationships that could be construed as a potential conflict of interest.
