# Supplementary material for: Risk and Prognosis of Secondary Bladder Cancer After Radiation Therapy for Rectal Cancer: A Large Population-Based Cohort Study
Source: Front Oncol. 2021 Jan 25;10:586401. doi: 10.3389/fonc.2020.586401 (PMC7868538; doi:10.3389/fonc.2020.586401)
Supplement: Supplementary file 1 [file DataSheet_1.docx]

**Supplementary Figures**


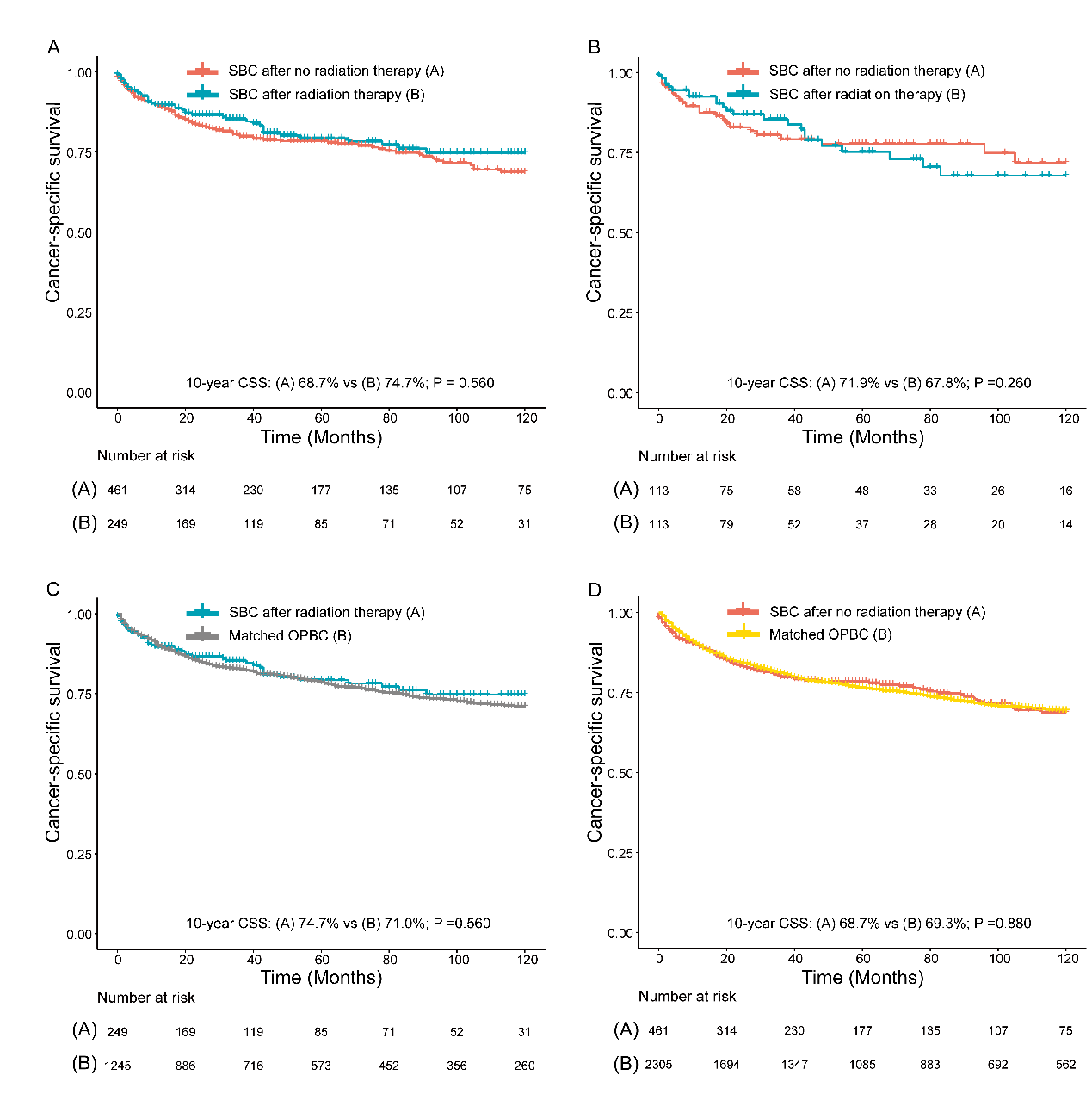


**Supplementary Fig 1.** (A) Survival comparison between rectal cancer (RC) patients who developed secondary bladder cancer (SBC) after RT and RC patients who developed SBC after no RT (NRT) (before PSM); (B) Survival comparison between RC patients who developed SBC after RT and RC patients who developed SBC after NRT (after PSM); (C) Survival comparison between RC patients who developed BC after RT and patients with only primary bladder cancer (OPBC); (D) Survival comparison between RC patients who developed BC after NRT and patients with OPBC.

**NOTE.** (B) RC patients who developed SBC after RT and RC patients who developed SBC after NRT were matched by PSM at a ratio of 1:1. (C) and (D) are case-control comparisons, RC patients who developed SBC (cases) versus patients with OPBC (controls), with a PSM ratio of 1:5 for SBC versus OPBC. The variables matched for PSM included age at SBC diagnosis, year of SBC diagnosis, race, stage of SBC and type of treatment for SBC. The detailed patient characteristics of OPBC before and after PSM are shown in the supplementary data. HRs were calculated using Cox regression.

**Abbreviations:** HR, hazard ratio; RC, rectal cancer; RT, radiation therapy; CI, confidence interval.

**Supplementary Tables**

**Supplementary Table 1.** Subgroup Analyses of Hazard Ratios of Developing Secondary Bladder Cancer in RC Patients who Received RT versus Those who did not Receive RT.

| Subgroup | RT (No. of events/total No.) | NRT (No. of events/total No.) | HR (95%CI) | P-value |
| --- | --- | --- | --- | --- |
| Sex (Female) | 53/9,493 | 97/23,140 | 1.46 (1.04-2.04) | 0.028 |
| Sex (Male) | 196/15,029 | 364/26,984 | 1.06 (0.89-1.26) | 0.530 |
| Age at RC diagnosis (20-49) | 17/4,211 | 15/4,921 | 1.63 (0.82-3.24) | 0.160 |
| Age at RC diagnosis (50-69) | 146/13,870 | 241/24,840 | 1.21 (0.98-1.48) | 0.075 |
| Age at RC diagnosis (70+) | 86/6,441 | 205/20,363 | 1.39 (1.08-1.79) | 0.010 |
| Year of RC diagnosis (1975-1984) | 39/2,604 | 140/14,092 | 1.51 (1.06-2.15) | 0.023 |
| Year of RC diagnosis (1985-1994) | 85/5,710 | 154/12,951 | 1.25 (0.96-1.64) | 0.093 |
| Year of RC diagnosis (1995-2004) | 92/7,500 | 127/11,772 | 1.14 (0.87-1.49) | 0.340 |
| Year of RC diagnosis (2005+) | 33/8,708 | 40/11,309 | 1.07 (0.67-1.69) | 0.780 |
| Race (White) | 227/20,365 | 432/41,826 | 1.19 (1.01-1.40) | 0.034 |
| Race (Black) | 8/1,659 | 14/3,555 | 1.28 (0.54-3.06) | 0.580 |
| Race (Other) | 14/2,498 | 15/4,744 | 1.93 (0.93-4.01) | 0.077 |
| Grade I/II | 184/17,921 | 314/34,238 | 1.23 (1.02-1.47) | 0.028 |
| Grade III/IV | 40/4,035 | 42/4,416 | 1.12 (0.72-1.73) | 0.610 |
| Stage (Localized) | 90/7,099 | 348/35,064 | 1.37 (1.08-1.72) | 0.008 |
| Stage (Regional) | 159/17,423 | 113/15,060 | 1.37 (1.08-1.75) | 0.011 |
| Histology (Adenocarcinoma) | 222/22,197 | 442/47,596 | 1.19 (1.01-1.39) | 0.039 |
| Histology (Mucous tumor) | 27/1,932 | 17/1,964 | 1.75 (0.95-3.21) | 0.072 |
| Tumor size (<2 cm) | 1/743 | 17/3,460 | 0.23 (0.03-1.70) | 0.150 |
| Tumor size (>2 cm) | 38/7,213 | 23/5,839 | 1.42 (0.84-2.38) | 0.190 |
| Chemotherapy (No) | 86/6,089 | 433/46,321 | 1.39 (1.10-1.75) | 0.005 |
| Chemotherapy (Yes) | 163/18,433 | 28/3,803 | 1.26 (0.84-1.88) | 0.260 |

**NOTE**. Fine-Gray competing risk regression analyses are used to calculate the hazard ratios (HRs) and 95% confidence intervals (CIs) for SBC in rectal cancer (RC) patients treated with radiation therapy (RT) versus patients not treated with RT.

**Abbreviations:** HR, hazard ratio; CI, confidence interval; RT, radiation therapy; NRT, no radiation therapy; RC, rectal cancer; BC, bladder cancer; SBC, second bladder cancer.

**Supplementary Table 2.** Patients Characteristics of Secondary Bladder Cancer Before and After PSM Matching.

| Characteristic | Surgery alone | | | Surgery with RT | | |
| --- | --- | --- | --- | --- | --- | --- |
|  | Surgery alone (n=461) | Surgery with RT (n=249) | P-value | Surgery alone (n=113) | Surgery with RT (n=113) | P-value |
| Age at RC diagnosis, No. (%), years |  |  | 0.004 |  |  | 0.211 |
| 20-49 | 0 (0) | 4 (1.6) |  | 0 (0) | 3 (2.6) |  |
| 50-69 | 97 (21.1) | 67 (26.9) |  | 26 (23.0) | 27 (23.9) |  |
| ≥ 70 | 364 (79.0) | 178 (71.5) |  | 87 (77.0) | 83 (73.5) |  |
| Year of RC diagnosis, No. (%) |  |  | <0.001 |  |  | 0.072 |
| 1975-1984 | 46 (10.0) | 7 (2.8) |  | 9 (8.0) | 6 (5.3) |  |
| 1985-1994 | 101 (21.9) | 43 (17.3) |  | 20 (17.7) | 37 (32.8) |  |
| 1995-2004 | 149 (32.3) | 71 (28.5) |  | 38 (33.6) | 33 (293) |  |
| ≥ 2005 | 165 (35.8) | 128 (51.4) |  | 46 (40.7) | 37 (32.6) |  |
| Sex, No. (%) |  |  | 1 |  |  | 0.060 |
| Female | 97 (21.0) | 53 (21.3) |  | 20 (17.7) | 33 (29.2) |  |
| Male | 364 (79.0) | 196 (78.7) |  | 93 (82.3) | 80 (70.8) |  |
| Race, No. (%) |  |  | 0.309 |  |  | 0.682 |
| White | 432 (93.7) | 227 (91.2) |  | 103 (91.2) | 99 (87.6) |  |
| Black | 14 (3.0) | 8 (3.2) |  | 4 (3.5) | 6 (5.3) |  |
| Other | 15 (3.3) | 14 (5.6) |  | 6 (5.3) | 8 (7.1) |  |
| Tumor grade, No. (%) |  |  | 0.067 |  |  | 0.118 |
| Grade I/II | 206 (44.7) | 116 (46.6) |  | 37 (32.8) | 44 (38.9) |  |
| Grade III/IV | 201 (43.6) | 91 (36.5) |  | 57 (50.4) | 42 (37.2) |  |
| Unknow | 54 (11.7) | 42 (16.9) |  | 19 (16.8) | 27 (23.9) |  |
| Tumor stage, No. (%) |  |  | 0.031 |  |  | 0.464 |
| Localized | 335 (72.7) | 177 (71.1) |  | 75 (66.4) | 82 (72.6) |  |
| Regional | 87 (18.9) | 35 (14.1) |  | 26 (23.0) | 23 (20.4) |  |
| Distant | 14 (3.0) | 15 (6.0) |  | 3 (2.6) | 4 (3.5) |  |
| Unstaged | 25 (5.4) | 22 (8.8) |  | 9 (8.0) | 4 (3.5) |  |
| Surgery, No. (%) |  |  | 0.568 |  |  | 1 |
| No | 34 (7.0) | 22 (8.5) |  | 10 (8.8) | 11 (9.7) |  |
| Yes | 427 (93.0) | 227 (91.5) |  | 103 (91.2) | 102 (90.3) |  |
| Chemotherapy, No. (%) |  |  | 0.757 |  |  | 0.859 |
| No | 387 (83.9) | 206 (82.7) |  | 93 (82.3) | 95 (84.1) |  |
| Yes | 74 (16.1) | 43 (17.3) |  | 20 (17.7) | 18 (15.9) |  |
| Radiation, No. (%) |  |  | 0.003 |  |  | 0.174 |
| No | 435 (94.4) | 247 (99.2) |  | 106 (93.8) | 111 (98.2) |  |
| Yes | 26 (5.6) | 2 (0.8) |  | 7 (6.2) | 2 (1.8) |  |

**NOTE.** Rectal cancer (RC) patients who developed SBC after radiation therapy (RT) were matched to patients after no RT(NRT), with a PSM ratio of 1:1. The variables matched for PSM included age at SBC diagnosis, year of SBC diagnosis, race, stage of SBC and type of SBC treatment.

**Abbreviations:** RC, rectal cancer; RT, radiation therapy; NRT, no radiation therapy; BC, bladder cancer.

**Supplementary Table 3.** Patients Characteristics of Secondary Bladder Cancer and Matched Primary Bladder Cancer.

| Characteristic | Surgery alone | | | Surgery with RT | | |
| --- | --- | --- | --- | --- | --- | --- |
|  | Surgery alone (n=461) | PSM (n=2,305) | P-value | Surgery with RT (n=249) | PSM (n=1,245) | P-value |
| Age at RC diagnosis, No. (%), years |  |  | 1 |  |  | 0.892 |
| 20-49 | 0 (0) | 0 (0) |  | 4 (1.6) | 20 (1.6) |  |
| 50-69 | 97 (21) | 482 (20.9) |  | 67 (26.9) | 317 (25.5) |  |
| ≥ 70 | 364 (79) | 1,823 (79.1) |  | 178 (71.5) | 908 (72.9) |  |
| Year of RC diagnosis, No. (%) |  |  | 0.413 |  |  | 0.214 |
| 1975-1984 | 46 (10.0) | 249 (10.8) |  | 7 (2.8) | 66 (5.3) |  |
| 1985-1994 | 101 (21.9) | 502 (21.8) |  | 43 (17.3) | 174 (14.0) |  |
| 1995-2004 | 149 (32.3) | 661 (28.7) |  | 71 (28.5) | 339 (27.2) |  |
| ≥ 2005 | 165 (35.8) | 893 (38.7) |  | 128 (51.4) | 666 (53.5) |  |
| Sex, No. (%) |  |  | 0.901 |  |  | 0.781 |
| Female | 97 (21.0) | 494 (21.4) |  | 53 (21.3) | 278 (22.3) |  |
| Male | 364 (79.0) | 1,811 (78.6) |  | 196 (78.7) | 967 (77.7) |  |
| Race, No. (%) |  |  | 0.433 |  |  | 0.537 |
| White | 432 (93.7) | 2,142 (92.9) |  | 227 (91.2) | 1,114 (89.5) |  |
| Black | 14 (3.0) | 98 (4.3) |  | 8 (3.2) | 60 (4.8) |  |
| Other | 15 (3.3) | 65 (2.8) |  | 14 (5.6) | 71 (5.7) |  |
| Tumor grade, No. (%) |  |  | 0.771 |  |  | 0.813 |
| Grade I/II | 206 (44.7) | 1,032 (44.8) |  | 116 (46.6) | 566 (45.5) |  |
| Grade III/IV | 201 (43.6) | 977 (42.4) |  | 91 (36.5) | 481 (38.6) |  |
| Unknow | 54 (11.7) | 296 (12.8) |  | 42 (16.9) | 198 (15.9) |  |
| Tumor stage, No. (%) |  |  | 0.953 |  |  | 0.715 |
| Localized | 335 (72.7) | 1,701 (73.8) |  | 177 (71.1) | 863 (69.3) |  |
| Regional | 87 (18.9) | 414 (18) |  | 35 (14.1) | 203 (16.3) |  |
| Distant | 14 (3.0) | 64 (2.8) |  | 15 (6.0) | 61 (4.9) |  |
| Unstaged | 25 (5.4) | 126 (5.4) |  | 22 (8.8) | 118 (9.5) |  |
| Surgery, No. (%) |  |  | 0.714 |  |  | 0.836 |
| No | 34 (7.4) | 159 (6.9) |  | 22 (8.8) | 105 (8.4) |  |
| Yes | 427 (92.6) | 2,146 (93.1) |  | 227 (91.2) | 1,140 (91.6) |  |
| Chemotherapy, No. (%) |  |  | 0.504 |  |  | 0.660 |
| No | 387 (83.9) | 1,966 (85.3) |  | 206 (82.7) | 1,047 (84.1) |  |
| Yes | 74 (16.1) | 339 (14.7) |  | 43 (17.3) | 198 (15.9) |  |
| Radiation, No. (%) |  |  | 0.971 |  |  | 0.615 |
| No | 435 (94.4) | 2,171 (94.2) |  | 247 (99.2) | 1,227 (98.6) |  |
| Yes | 26 (5.6) | 134 (5.8) |  | 2 (0.8) | 18 (1.4) |  |

**NOTE.** Rectal cancer (RC) patients who developed second bladder cancer (SBC) were matched with patients with only primary bladder cancer (OPBC) at a PSM ratio of 1:5 for other SBC versus OPBC. The matched variables for PSM included age at SBC diagnosis, year of SBC diagnosis, race, stage of SBC and type of SBC treatment.

**Abbreviations:** RC, rectal cancer; RT, radiation therapy; NRT, no radiation therapy; BC, bladder cancer; SBC, second bladder cancer; OPBC, only primary bladder cancer.
